# Integrin-linked kinase activity modulates the pro-metastatic behavior of ovarian cancer cells

**DOI:** 10.18632/oncotarget.7880

**Published:** 2016-03-03

**Authors:** Lana Bruney, Yueying Liu, Anne Grisoli, Matthew J. Ravosa, M. Sharon Stack

**Affiliations:** ^1^ Department of Medical Physiology & Pharmacology, University of Missouri School of Medicine, Columbia, MO, USA; ^2^ Harper Cancer Research Institute, University of Notre Dame, South Bend, IN, USA; ^3^ Departments of Chemistry & Biochemistry and University of Notre Dame, Notre Dame, IN, USA; ^4^ Biological Sciences, University of Notre Dame, Notre Dame, IN, USA

**Keywords:** integrin linked kinase, ovarian cancer, integrin, membrane type 1 matrix metalloproteinase, metastasis

## Abstract

Epithelial ovarian cancer (EOC) is the most fatal gynecologic cancer in the U.S., resulting in >14,000 deaths/year. Most women are diagnosed at late stage with widely disseminated intra-peritoneal metastatic disease, resulting in a 5-year survival rate of <30%. EOCs spread via direct extension and exfoliation into the peritoneal cavity, adhesion to peritoneal mesothelial cells, mesothelial cell retraction to expose sub-mseothelial matrix and anchoring in the type I collagen-rich matrix to generate secondary lesions. As a molecular-level understanding of EOC metastasis may identify novel therapeutic targets, the current study evaluated the expression and activity of integrin-linked kinase (ILK), a Ser/Thr protein kinase activated upon integrin-mediated adhesion. Results show that ILK is co-expressed in EOC with the pro-metastatic enzyme membrane type 1 matrix metalloproteinase (MT1-MMP) and catalyzed phosphorylation of the cytoplasmic tail of the proteinase. Downregulation of ILK expression or activity reduced adhesion to and invasion of collagen gels and organotypic meso-mimetic cultures. As an initial early event in EOC metastasis is integrin-mediated adhesion, these results suggest that further evaluation of ILK inhibitors as anti-metastatic agents in EOC is warranted.

## INTRODUCTION

Epithelial ovarian cancer (EOC) is one of the most common gynecologic malignancies, generally developing in women over the age of forty [1, 2]. EOC symptoms are usually vague, and with no sufficiently accurate population-level screening test currently available, early diagnosis is often difficult. When women with EOC are diagnosed prior to metastatic dissemination, the overall 5-year survival rate is 92%; however, nearly 85% of women are diagnosed with metastasis already present, decreasing the survival rate to less than 30%. As mortality is directly related to the prevalence of metastatic disease, a molecular-level understanding of EOC metastasis is warranted.

Ovarian cancers spread predominantly through direct extension into the peritoneal cavity and by detachment or exfoliation from primary tumor on the ovary or fallopian tube, facilitating intra-peritoneal dissemination. Metastasizing cells initially adhere to the single layer of mesothelial cells lining the peritoneal cavity, whereupon they induce mesothelial cell retraction and exposure of the underlying extracellular matrix (ECM) to enable metastatic anchoring in the interstitial type I collagen-rich sub-mesothelial matrix [3]. Indeed, both *in vitro* studies and micrographs of excised human peritoneum-associated tumors have shown that the metastases are attached to connective tissue directly beneath the mesothelial cell layer (sub-mesothelial ECM) and that mesothelial cells are not present directly under the tumor mass. Metastases are most commonly found within the omentum, the peritoneum, the diaphragm, and bowel surfaces [4]. This complex process of detachment, adhesion, and sub-mesothelial anchoring provides multiple potential targets for therapeutic intervention.

Adhesion of EOC cells to the sub-mesothelial collagen matrix is mediated by integrins, a family of transmembrane glycoproteins which regulate many cell-cell and cell-matrix adhesive interactions [5-8]. Integrins are comprised of a functionally linked α and β subunit, the cytoplasmic domains of which make important contributions to various aspects of overall integrin function [9-11]. In ovarian tissues, α2, α3, α_v_, β1 and β3 integrin subunits are highly expressed [12-14]. Expression of β1 integrin has been directly correlated with shorter overall survival in women with EOC [15]. Moreover, cDNA microarray studies have shown that β1 integrin engagement also regulates expression of multiple gene products that contribute to metastatic successes, specifically membrane type 1 matrix metalloproteinase (MT1-MMP, MMP14) [7, 16].

A yeast two-hybrid screen identified integrin-linked kinase (ILK) as a binding partner of the cytoplasmic domains of β1 and β3 integrins [17-18]. ILK is a serine/threonine protein kinase that regulates integrin-mediated cell adhesion and provides a molecular scaffold for the assembly of signaling molecules, physically linking ECM proteins and growth factors via integrins and receptor tyrosine kinases to the actin cytoskeleton [19-20]. Cellular processes facilitated by ILK activity include integrin relocation to focal adhesion sites, increased invasion of ECM, decreased cell-cell adhesion, and the suppression of anoikis [20-21]. ILK activity is positively regulated in a PI3K-dependent manner by both cell-ECM interactions and growth factor receptors [21-23]. Once activated, ILK directly phosphorylates several key signaling molecules, including protein kinase B (Akt) at Ser^473^ and glycogen synthase kinase 3β (GSK3β), to affect cell survival, cell cycle, cell adhesion and ECM modification [24]. Previous studies have shown that ILK expression is enhanced in advanced ovarian tumors compared to benign ovarian tumors and normal ovarian epithelium [25]. Inhibition of ILK has been shown to induce apoptosis and cell cycle arrest, making ILK an attractive therapeutic target for cancer treatment [26].

Many of the extracellular events responsible for the regulation of metastatic cell behavior occur at the cell membrane and are controlled by pericellular proteolysis. Matrix metalloproteinases (MMPs) are zinc-dependent extracellular matrix degrading proteases that facilitate proteolysis at the cell surface to directly influence cell behavior [27-31]. Enhanced expression of MMPs is crucial to tissue remodeling and has been widely implicated in tumor invasion, metastasis, and angiogenesis [27-32]. These observations have prompted considerable interest in utilizing MMP inhibitors in large scale cancer clinical trials, but most have shown limited efficacy and treatment failure, attributed to the broad spectrum nature of most compounds [33-34]. Membrane type 1 matrix metalloproteinase (MT1-MMP or MMP-14) is a transmembrane collagenase that is not detected in normal ovarian surface epithelium nor in benign ovarian tumors, but is widely expressed in ovarian carcinomas of all histologic types, with enhanced expression in metastases relative to primary tumors [16, 35-37]. High expression of MT1-MMP has been linked with decreased survival in EOC patients [38-36]. MT1-MMP activity at the EOC cell surface is implicated in a number of pro-metastatic events, including induction of cell-matrix detachment and metastatic shed, promotion of multi-cellular aggregate formation, invasion of the collagen-rich sub-mesothelial matrix, and proliferation within a physically constrained 3-dimensional matrix microenvironment [6, 39-41]. The MT1-MMP transmembrane domain consists of a stretch of hydrophobic amino acids that traverse the cell membrane, and a short (20 amino acid) cytoplasmic tail [42]. Localization of active MT1-MMP to the plasma membrane facilitates modification of the pericellular micro-environment. In addition to control of proteinase activity via inhibitors, MT1-MMP activity at the cell surface is regulated through phosphorylation of its cytoplasmic tail and numerous studies have demonstrated cytoplasmic tail involvement in intracellular signaling, intermolecular interactions, and cellular responses [40, 41, 43, 44]. The objective of the current study was to evaluate the expression of ILK and MT1-MMP in ovarian cancer cells and tissues and to examine the effect of ILK inhibition on pro-metastatic ovarian cancer cell behavior.

## RESULTS

### ILK and MT1-MMP are co-expressed in human ovarian tumor tissues and cells

**Figure 1:**
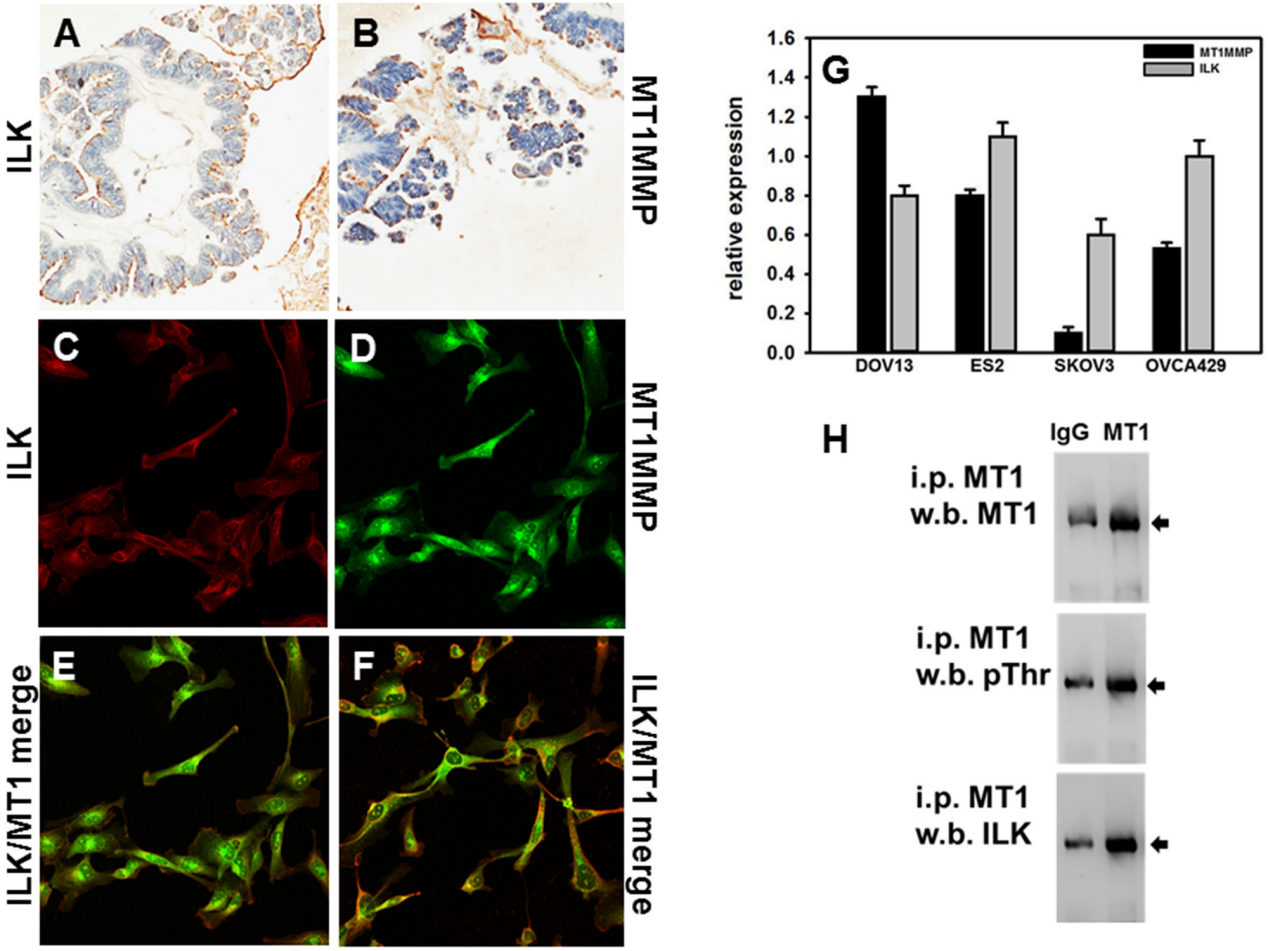
Expression of ILK and MT1-MMP in human ovarian carcinoma tissues and cells **A, B.** Microarrayed cores of ovarian adenocarcinoma were subjected to immunohistochemical analyses for ILK (A) or MT1-MMP (B) as described in Materials and Methods. 20x magnification. **C-F.** Fluorescence co-localization of ILK and MT1-MMP in DOV13 human ovarian cancer cells. Subconfluent monolayers of cells were seeded onto collagen type I coated coverslips and processed for immunofluorescence. (C) ILK (red), (D) MT1-MMP (green), (E, F) representative merged images. **G.** Quantitative real time PCR analysis of ILK and MT1-MMP in ovarian cancer cells. The comparative C_T_ method was used to determine average relative quantitation. Results represent the mean of a minimum of three independent experiments. **H.** Immunoprecipitation of MT1-MMP. Cell lysates were subjected to immunoprecipitation with anti-MT1-MMP antibody (designated ‘MT1’) or control IgG and protein G beads. Immunoprecipitates were electrophoresed on 9% polyacrylamide gels and subject to western blotting using anti-MT1-MMP (upper panel), anti-phospho-T*X*R antibodies (middle panel) or anti-ILK antibodies (lower panel), followed by secondary antibodies and enhanced chemiluminescent detection.

It has previously been reported that expression of ILK correlates with the progression of ovarian cancer, with enhanced expression found in high-grade human tumors compared to weak staining in low-grade lesions [25]. To determine whether ILK and MT1-MMP immunoreactivity are similarly localized in ovarian cancer, serial sections of a tumor tissue microarray were subjected to IHC analysis for either ILK or MT1-MMP. Representative examples of positive staining are shown in Figure 1A and 1B, respectively. From this TMA, 39% of the cores stained positively for ILK (graded as ‘moderate’ or ‘strong’). Of the ILK positive cores, 53% also stained positively for MT1-MMP. Co-expression of ILK and MT1-MMP was confirmed in a panel of ovarian cancer cell lines using real time quantitative PCR analysis (Figure 1G). As DOV13 cells exhibited the highest expression of MT1-MMP, immunofluorescence microscopy was used to examine protein expression. As shown in Figure 1C-1F, both ILK and MT1-MMP proteins were expressed, with co-localization observed at various points on the cell membrane. Co-localization of ILK and MT1-MMP, together with previous studies showing phosphorylation of Thr^567^ of the MT1-MMP cytoplasmic tail [40, 41, 43], suggest that MT1-MMP may be an ILK substrate. To examine this possibility, cell lysates were immuno-precipitated with antibodies directed against MT1-MMP and the resulting precipitates probed with antibodies directed against MT1-MMP (Figure 1H upper panel), phospho-Thr (Figure 1H middle panel) or ILK (Figure 1H, lower panel). Results show the presence of MT1-MMP/ILK complexes containing Thr-phosphorylated MT1-MMP.

### Modulation of ILK expression and/or activity alters ovarian cancer cell behavior

**Figure 2:**
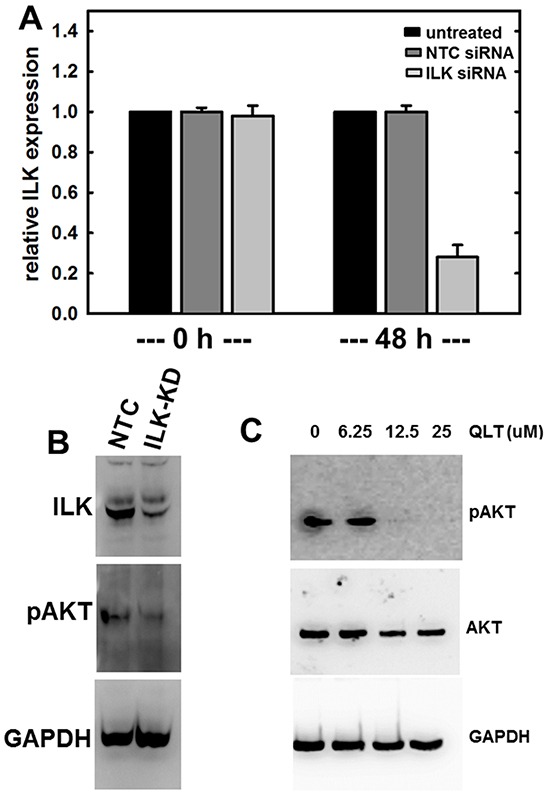
Regulation of ILK expression and activity **A.** siRNA-mediated silencing of ILK expression as confirmed using quantitative real time PCR analysis of ILK levels as described in Materials and Methods. Results are expressed relative to non-targeting control siRNA (NTC) and represent the mean of a minimum of three independent experiments. A Mann-Whitney U test was employed to determine statistical significance between NTC siRNA and ILK-siRNA as indicated. No difference between untreated and NTC siRNA was detected at any time point (0 h, p=0.479; 48 h, p=0.487). A significant difference is observed comparing either untreated or NTC-siRNA with ILK-siRNA at 48 h (p<.001 in both cases). **B.** Western blot analysis showing expression of ILK (upper panel) in cells transfected with NTC-siRNA or ILK-siRNA as indicated. In the middle panel, cell lysate was probed with the ILK downstream mediator phospho-AKT-Ser^473^. Lower panel is probed with GAPDH as a loading control. **C.** Western blot analysis showing inhibition of ILK activity using QLT0267, as determined by evaluating phosphorylation of AKT. Cells were treated with the ILK inhibitor QLT0267 or vehicle control, as indicated. Cell lysates were probed with antibodies directed against phospho-AKT-Ser473 (upper panel), total AKT (middle panel), or GAPDH (lower panel) as indicated.

To examine the contribution of ILK to cellular events that are impacted by MT1-MMP, ILK expression and/or activity were modulated. Initial attempts to overexpress wild-type and constitutively active ILK resulted in significant cellular toxicity (not shown). As an alternative approach, siRNA-based silencing of ILK expression and small molecule inhibition of ILK activity were utilized. Real time quantitative PCR analysis revealed significant suppression of ILK mRNA in cells transfected with ILK siRNA compared to non-targeted siRNA control (NTC-siRNA) at 24 hours (*p=*0.050, data not shown) and 48 hours (Figure 2A). Down-regulation at the protein level was also demonstrated via western blot analysis (Figure 2B, top panel). Loss of ILK was further confirmed by examining phosphorylation of AKT in cells plated on type I collagen as a surrogate measure of ILK kinase activity. Reduced levels of pAkt-Ser^473^ are apparent when ILK is silenced using siRNA (Figure 2B, center panel). It should be noted that non-transfected control cells and NTC-siRNA cells behaved interchangeably throughout these studies. Similarly, treatment of cells with the ILK inhibitor QLT0267 also blocks AKT-Ser^473^ phosphorylation in a dose-dependent manner (Figure 2C).

**Figure 3:**
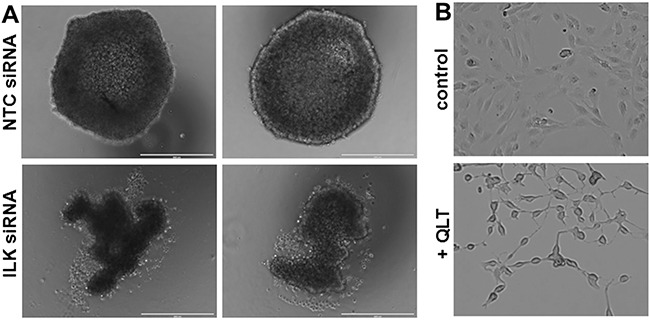
Reduced ILK modulates MCA formation and cell spreading **A.** MCAs were generated using the hanging drop method as described in Materials and Methods. Representative examples of two MCAs formed from NTC-siRNA control cells (control, upper panels) and ILK-knockdown cells (ILK-siRNA, lower panels) are shown. Scale bar, 400 um. **B.** Cells were plated on coverslips and incubated with vehicle (control, upper panel) or the ILK inhibitor QLT0267 (+QLT, lower panel, 25 uM) for 12 h prior to visualization with light microscopy.

Multicellular aggregates (MCAs) function as a key metastatic unit for ovarian cancer and we have previously shown that ectopic or endogenous MT1-MMP expression leads to the formation of larger MCAs [40]. Comparison of MCAs formed by control cells or cells transfected with ILK siRNA show that downregulation of ILK expression disrupts MCA integrity, resulting in the formation of less cohesive MCAs (Figure 3A). Morphometric analysis of changes in MCA formation were quantitatively assessed by measuring the length (l) and width (w) of MCAs. Whereas control MCAs are quite spherical (l/w = 1.05 +/− 0.02), MCAs formed from cells with ILK knockdown formed ellipsoids (l/w = 1.23 +/− 0.04). These results are consistent with previous data showing that MCAs form less efficiently when cells express a phospho-defective variant of MT1-MMP (T567A mutant) [40] and suggest that ILK-catalyzed MT1-MMP phosphorylation may modulate MCA dynamics. This effect is likely related to the inability of cells to spread efficiently in the absence of ILK activity, as shown in control experiments using cells treated with the ILK inhibitor QLT0267 (Figure 3B). These data show that while cell length remains similar, cells appear with elongated projections with very little cytoplasmic spreading.

**Figure 4:**
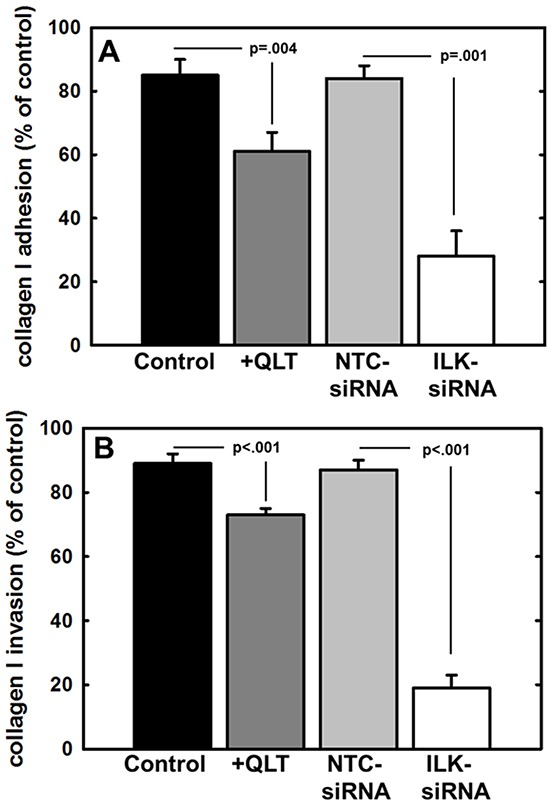
Effect of modified ILK expression and activity on adhesion to and invasion of type I collagen **A.** Adhesion to type I collagen. Untreated control cells (control, black bar), cells treated with QLT0267 (25 uM, dark grey bar), cells transfected with non-targeting control siRNA (NTC-siRNA, light grey bar) or cells transfected with ILK-targeting siRNA (ILK-siRNA, white bar) were incubated in wells coated with type I collagen for 30 min prior to washing to remove unbound cells and quantitation of adherent cells. Results are depicted as the percentage of total cells seeded allowed to adhere overnight and represent the mean of three independent experiments. Statistical significance was determined using Mann-Whitney U tests and p values are indicated. **B.** Invasion of 3-dimensional collagen gels. Cells, defined as in (A), were seeded into transwells containing a 8 um pore filter overlaid with a 3-dimensional collagen gel as described in Materials and Methods. After incubation for 24 h, non-invading cells were scraped from the top of the filter, the filter was removed and stained. Invading cells adherent to the underside of the filter were enumerated. Results represent the percentage of cells migrated relative to total cells seeded and represent the mean of three independent experiments. Statistical significance was determined using Mann-Whitney U tests and p values are indicated.

ILK activity is stimulated by adhesion to the extracellular matrix [45] and ILK has been shown to regulate integrin-mediated cell adhesion, migration, and cytoskeletal reorganization. As collagen type I is the most abundant matrix protein in the sub-mesothelial stroma and is the preferred substrate for β1 integrin-mediated ovarian cell attachment during peritoneal metastasis [5-8, 46], the role of ILK in the attachment to and invasion of collagen type I was assessed. To evaluate adhesion to collagen, cells were incubated atop a collagen type I matrix, washed to remove non-adherent cells, and adherent cells were enumerated. Relative to controls, adhesion was significantly reduced by inhibition of ILK activity using the small molecule inhibitor QLT0267 or by downregulation of ILK expression using siRNA (Figure 4A). Our lab and others have previously shown that MT1-MMP activity is necessary to support the type I collagen invasive phenotype by ovarian cancer cells [5, 16, 37, 40]. To examine the contribution of ILK activity to this process, cells were incubated in a transwell chamber containing a type I collagen gel and cells migrating through the 3-dimensional gel to the underside of the collagen coated membrane were enumerated. Inhibition of both ILK activity and ILK expression significantly impacted collagen invasive activity relative to parental cells (control) or cells transfected with a non-targeting control siRNA (NTC-siRNA) (Figure 4B). Cell viability was not altered by incubation for 24 h with the ILK inhibitor (not shown).

**Figure 5:**
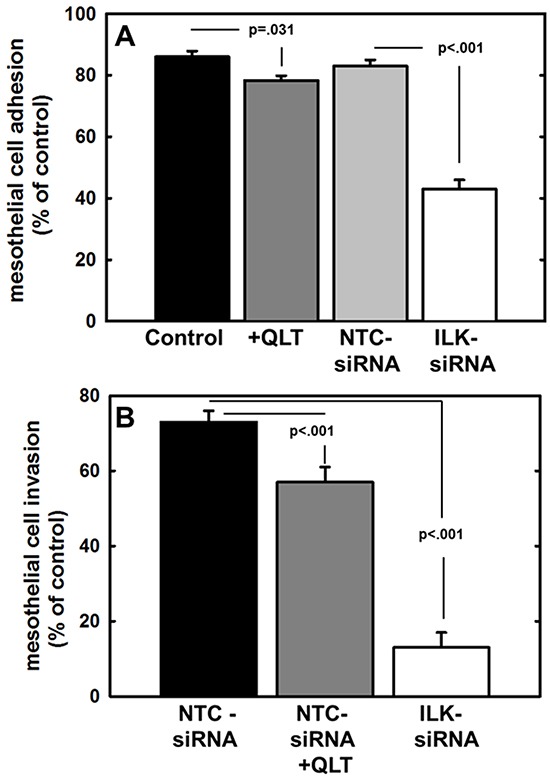
Effect of modified ILK expression and activity on adhesion to and invasion of live mesothelial cell monolayers or meso-mimetic cultures **A.** Adhesion to mesothelial monolayers. Untreated control cells (control, black bar), cells treated with QLT0267 (25 uM, dark grey bar), cells transfected with non-targeting control siRNA (NTC-siRNA, light grey bar) or cells transfected with ILK-targeting siRNA (ILK-siRNA, white bar) were fluorescently labeled with CMFDA and incubated in wells containing a confluent monolayer of LP9 human peritoneal mesothelial cells for 30 min prior to washing to remove unbound cells and quantitation of adherent cells. Results are depicted as the percentage of total cells seeded allowed to adhere overnight and represent the mean of three independent experiments. Statistical significance was determined using Mann-Whitney U tests and p values are indicated. **B.** Invasion of meso-mimetic cultures. Control cells transfected with non-targeting siRNA (NTC-siRNA, black bar), NTC-siRNA control cells treated with QLT0267 (NTC-siRNA+QLT, dark grey bar), or cells transfected with ILK-targeting siRNA (ILK-siRNA, white bar) were fluorescently labeled with CMFDA and incubated in transwells containing organotypic meso-mimetic cultures comprised of a confluent layer of LP9 human peritoneal mesothelial cells cultured atop a 3-dimensional type I collagen gel on a porous membrane [57-58]. After incubation for 24 h, cells were scraped from the top of the filter, the filter was removed and stained. Invading cells adherent to the underside of the filter were enumerated. Results represent the percentage of cells migrated relative to total cells seeded and represent the mean of three independent experiments. A Kruskal-Wallis test was used to find a significant mean difference among all three groups (p=0.007). Statistical significance between 2 groups was determined using Mann-Whitney U tests and p values are indicated.

An initial early event in ovarian cancer metastasis is the adhesion of tumor cells to peritoneal mesothelial cells, leading to retraction of the mesothelial monolayer and exposure of the submesothelial ECM [3, 4, 47]. This process has been shown to involve β1 integrin mediated cell-cell adhesion [7-8]. To evaluate the effect of ILK silencing on ovarian cancer cell attachment to live meso-mimetic organotypic cultures, human mesothelial cells (LP9) were plated atop a collagen type I matrix and grown to confluency. Fluorescently labeled EOC cells were then seeded atop this mesothelial monolayer, incubated for 30 minutes, washed to remove non-adherent cells, and adherent cells were enumerated. Inhibition of ILK activity or expression significantly reduced ovarian cancer cell adhesion to the meso-mimetics (Figure 5A). MT1-MMP activity enhances ovarian cancer cell penetration of meso-mimetic cultures [48]; however the effect of ILK on this process has not been previously examined. Using live meso-mimetic cultures generated atop 8μm porous membranes seated in the upper compartment of a transwell insert, invasive activity was evaluated. Reduction of ILK activity with QLT0267 or ILK expression with siRNA significantly inhibited ovarian cancer cell invasion of meso-mimetic cultures (Figure 5B).

## DISCUSSION

A more detailed understanding of the molecular mechanisms integral to early events in ovarian cancer metastasis, namely adhesion and invasion, is key to creating successful anti-metastatic therapeutics. MT1-MMP, an extracellular matrix degrading protease localized to the cell surface, has been implicated in invasion and metastasis of a number of human tumor cells [6, 39-42]. MT1-MMP has not been detected in normal OSE nor in benign ovarian tumors, but is widely expressed in ovarian carcinomas, where its enzymatic activity is key in promoting the migration to and invasion of sub-mesothelial collagen matrices [5, 16, 35, 38]. There is evidence to suggest that MT1-MMP activity is regulated through phosphorylation of Thr^567^ on its cytoplasmic tail [40, 41]. Expression and activity of ILK, a serine/threonine kinase activated by integrin-mediated cell adhesion, is increased in several different cancer types [49]. Overexpression and constitutive ILK activation promotes tumor formation in transgenic mice and also provokes oncogenic cell transformation into anchorage-independent, highly migratory and invasive cells [50]. In ovarian cancer, studies have shown up-regulated ILK expression compared to benign tumors and normal ovarian epithelium; additionally, a direct relationship between ILK expression and ovarian tumor grade has been demonstrated [25]. EOC metastatic implantation is initiated via intraperitoneal adhesive events resulting from cell-cell and cell-matrix interaction to mesothelial cells and tissues. β1 integrin-mediated adhesion has been implicated as a key event in this process and ILK is activated by β1 integrin adhesion, resulting in phosphorylation of cytoplasmic substrates that regulate key cellular processes. The current data support MT1-MMP as an additional ILK substrate and show that modulation of ILK expression and activity inhibit MT1-MMP-related pro-metastatic behaviors of ovarian cancer cells.

It has previously been reported that ILK is expressed in advanced ovarian cancers [25]. Our data confirm positive immunoreactivity for ILK in a subset of human ovarian tumor tissues. The staining of tumor tissue microarray serial sections for MT1-MMP revealed similar patterns of expression, with positive MT1-MMP immunoreactivity in approximately half of all tissues with positive ILK expression. Evaluation of ILK and MT1-MMP mRNA levels in ovarian cancer cells supported the co-expression of ILK and MT1-MMP. Fluorescence co-localization of the proteins at the cell membrane and co-immunoprecipitation of Thr-phosphorylated MT1-MMP with ILK provide additional support for the hypothesis that ILK-MT1-MMP interactions may regulate ovarian cancer cell behavior. Indeed, similarly enhanced invasive activity was observed using MDA-MB-231 cells transfected to express a phospho-mimetic T567E mutant of MT1-MMP [41]. Results of the current study show that knockdown or pharmacologic inhibition of ILK in ovarian cancer cells also modulates MCA dynamics, inhibits adhesion to collagen and mesothelial cells, and blocks invasion of 3-dimensional collagen gels as well as organotypic meso-mimetic cultures. These data support ILK as a potential therapeutic target to impede ovarian cancer metastasis. It is currently unclear why ILK silencing resulted in larger effects on adhesion and invasion relative to ILK inhibitor treatment, but this could possibly be related to issues of inhibitor stability in aqueous solution. It should also be noted that, based on the current data, we can not rule out the possibility that Ser/Thr kinases in addition to ILK may phosphorylate MT1-MMP. For example, it has previously been reported that phorbol ester-stimulated activation of protein kinase C induced Thr567 phosphorylation of MT1-MMP, impacting the migration and invasion of fibrosarcoma cells [51]. Additional Ser/Thr kinases including Akt, GSK3β, and MEK are also prevalent in the ovarian tumor microenvironment and are activated by a variety of stimuli, raising the possibility that reversible phosphorylation of MT1-MMP may function as a control mechanism to regulate proteinase-dependent cellular functions.

Together with the results of the current study, recent reports demonstrate the potential efficacy of ILK inhibition in ovarian cancer. ILK expression was shown to be enhanced downstream of endothelin-1/endothelin A receptor signaling and inhibition of this signaling axis reduced tumor growth [52]. Silencing of ILK expression using shRNA in ovarian cancer cells suppressed anti-apoptotic bcl-2 and increased pro-apoptotic bax expression, leading to enhanced apoptosis [53]. Depletion of ILK also delayed tumor formation in sub-cutaneous ovarian cancer xenograft models [50, 54]; however intra-peritoneal tumor growth was not evaluated. In a breast cancer model, pharmacologic inhibition of ILK with QLT0267 was shown to be synergistic with docetaxel, demonstrating enhanced cytotoxic activity, significantly reduced tumor growth, and enhanced survival compared to either agent alone [55]. These results suggest that further evaluation of ILK inhibitors, either alone or in combination with standard chemotherapeutic agents, is warranted in intra-peritoneal xenograft models of ovarian cancer metastasis.

## MATERIALS AND METHODS

### Materials

Anti-ILK antibodies were purchased from Millipore (Billerica, MA) (for Western blotting), Abcam (Cambridge, MA) (for immunoprecipation), or Sigma-Aldrich (St. Louis, MO) (for immunofluorescence). Glyceraldehyde-3-phosphate dehydrogenase (GAPDH) antibodies were purchased from Santa Cruz Biotechnology, Inc (Santa Cruz, CA). Antibodies directed against phospho-Akt(Ser473), total Akt, and phospho-Threonine (in the sequence phospho-Threonine-X-Arginine) were purchased from Cell Signaling Technology (Beverly, MA). MT1-MMP antibodies were purchased from Epitomics (Burlingame, CA). Rat tail collagen type I was purchased from BD Biosciences (San Diego, CA). Cell culture media and additives were purchased from Gibco Invitrogen (Carlsbad, CA) unless otherwise stated. TaqMan materials (TaqMan Fast Universal PCR Master Mix and TaqMan Gene Expression Assay Mix) were purchased from Life Technologies, Inc (Carlsbad, CA). The EDTA-free Complete Protease Inhibitor and Halt Phosphatase Inhibitor cocktails used in modified RIPA buffer were purchased from Roche Diagnostics (Indianapolis, IN) and Thermo Scientific (Rockford, IL), respectively. Protein G Sepharose beads used in immunoprecipitation studies were purchased from Sigma-Aldrich (St. Louis, MO). Immobilon-P polyvinylidene membranes were purchased from Millipore (Billerica, MA). SuperSignal West Dura Chemiluminescent Substrate was purchased from Thermo Scientific (Rockford, IL). ILK inhibitor QLT0267 was a generous gift from Dr. Shoukat Dedhar (BC Cancer Research Centre, Vancouver, B. C.). All chemicals were analytical grade.

### Cell lines and cell culture

Cells (DOV13, OVCA433, SKOV3 and ES2) were maintained in a humidified incubator under standard conditions (37°C with 5% CO_2_). Ovarian cancer cell lines DOV13 and OVCA433 were generously provided by Dr. Robert Bast, Jr. (M.D. Anderson Cancer Center, Houston, TX). ES2 and SKOV3 cells were obtained from ATCC. Cells were maintained in minimum essential media (MEM) supplemented with 10% fetal bovine serum (FBS), 1mM sodium pyruvate, 1mM non-essential amino acid, 50U/ml penicillin, and 50μg/ml streptomycin; DOV13 medium required an addition of 10μg/ml human recombinant insulin (Life Technologies, Inc., Carlsbad, CA). The human peritoneal mesothelial cell line LP9 was obtained from Coriell Aging Cell Repository (Coriell Institute, Camden, NJ). The LP9 cell line was maintained in 1:1 ratio Medium 199 and Ham's F12, supplemented with 15% FBS, 2mM glutamine, 1mM HEPES, 20mg/ml epidermal growth factor (R&D systems, Minneapolis, MN), 0.4μg/ml hydrocortisone, and 50U/ml penicillin, and 50μg/ml streptomycin.

Knockdown of ILK was completed using the ILK-targeted TriFECTa™ Dicer-Substrate RNAi kit (Integrated DNA Technologies, San Diego, CA) and Lipofectamine^®^ RNAiMAX transfection reagent (Life Technologies, Inc., Carlsbad, CA) according to manufacturers' protocol. Briefly, a transfection cocktail (Opti-MEM, Lipofectamine^®^ RNAiMAX and either ILK siRNA duplex or included non-targeting control siRNA) was placed in a cell culture vessel and incubated at room temperature for 5 minutes. Subconfluent DOV13 cells were trypsinized, neutralized, suspended in antibiotic-free media, and diluted to 2.5×10^5^ cells/ml. Upon incubation termination, 1ml of cell suspension was added to transfection cocktail and the entire solution incubated for 24 hours. After initial incubation, samples were again subjected to fresh transfection cocktail for an additional 24 hours. After this final incubation, samples were assessed to confirm knockdown and utilized immediately as described. For pharmacologic inhibition of ILK, cells were incubated with the ILK inhibitor QLT0267 (dissolved in DMSO) at the indicated doses in serum-free media, or with vehicle-only control.

### Western blotting and immunoprecipitation

Whole cell lysates from attached cultures were collected by the following process: the cell monolayer was washed twice with phosphate-buffered saline, treated with 600μl modified RIPA lysis buffer (150mM NaCl; 50mM Tris, pH 7.5; 20mM NaF; 10mM Na_2_P_2_O_7_; 5mM EDTA; 1% Triton X-100; 0.1% SDS) containing protease inhibitor and/or phosphatase inhibitor cocktails as appropriate, for 10 minutes at 4°C, scraped for complete collection, and then passed through a 26⅝ gauge syringe 5 times. Protein concentration was measured using DC™ Protein Assay (Bio-Rad Laboratories, Inc., Hercules, CA). For western blotting of whole cell lysates, lysates were collected as described above and protein content standardized. Protein (20μg) was electrophoresed on 9% SDS-polyacrylamide gels and transferred to methanol-activated polyvinylidene membranes. After transfer, membranes were incubated with 3% BSA in TBST (150mM NaCl, 25mM Tris, 0.05% Tween 20) to block non-specific binding for 1 hour at room temperature. Primary antibodies were diluted as indicated in 3% BSA/TBST and incubated overnight at 4°C. After washing, the membranes were incubated with horseradish peroxidase-conjugated secondary antibodies for 1 hour at room temperature, and then visualized with enhanced chemiluminescence using ImageQuant™ LAS4000 (GE Healthcare Life Sciences, Pittsburgh, PA). For assessment of controls, membranes were stripped of antibody by washing for 1 hour at room temperature in an optimized stripping buffer (150mM NaCl; 100mM β-mercaptoethanol; 50mM Tris, pH 6.8; 1% SDS; 0.02% NaN_3_), and then re-probed as appropriate for the control antibody. Samples were normalized to control antibody (GAPDH) and quantified using Multigauge v.2 for densitometric analysis (FUJIFILM, Tokyo, Japan). The migration positions of proteins were evaluated for consistency with reported molecular weights as determined using the formula: R_f_ = migration of protein/migration of dye front relative to a comparison the migration position of molecular weight standards plotted as Log Mwt vs R_f_. Immunoprecipitation was performed as previously described [41].

### Quantitative real time PCR (qPCR)

RNA was extracted from 10^6^ cells (as indicated) using RNeasy Mini Kit (Qiagen, Valencia, CA) in accordance with the manufacturer's instructions. cDNA was synthesized from 1-5μg of total RNA using RT^2^ First Strand Kit (Qiagen, Valencia, CA). Real time PCR was performed on a StepOnePlus™ Real-Time PCR System (Applied Biosystems, Foster City, CA). Detection of ILK was achieved using a specific Taqman Gene Expression Assay (Hs00199714_m1, Applied Biosystems, Foster City, CA) and conditions for amplification were as recommended by Applied Biosystems: an initial denaturation for 20 seconds at 95°C followed by 40 cycles of 95°C for 1 second and 60°C for 20 seconds. Taqman assay for housekeeping gene hypoxanthine ribosyltransferase (HPRT, Hs99999909_m1, Applied Biosystems, Foster City, CA) was used for normalization. Conditions for amplification using the iTaq SYBR Green Supermix (Bio-Rad Laboratories, Inc., Hercules, CA) as a fluorescent reporter were as follows: an initial denaturation for 10 min at 95°C followed by 40 cycles of 95°C for 15s and 60°C for 30s. PCR primer specificity was determined via melting curves, where products were heated at 95°C, cooled to 65°C, and then slowly melted at 0.5°C/s up to 95°C. In some experiments GAPDH was used as an internal control in each reaction (Applied Biosystems, Foster City, CA). The comparative C_T_ method (2^−(ΔC_t_sample - ΔC_t_control)^) was used to determine average relative quantitation.

### Immunohistochemistry and immunofluorescence

Immunohistochemical detection of antigen content in malignant ovarian tissue was performed using commercially available tissue microarrays containing 46 cores of ovarian adenocarcinoma (Biomax 110118) (US Biomax, Rockville, MD). Of the cores evaluated, 39 were serous adenocarcinoma, 2 mucinous adenocarcinoma, 2 endometrioid adenocarcinoma, and 3 clear cell carcinoma. The TMAs were de-parrafinized in xylene for 5 minutes (thrice) and soaked in absolute alcohol for 3 minutes. To inhibit endogenous peroxidase, slides were incubated for 30 minutes in 3.3% H_2_O_2_in methanol. Antigen retrieval was accomplished by incubation in heated sodium citrate (10mM) for 1 hour. Slides were processed using VECTASTAIN Elite ABC kit (Vector Laboratories, Burlingame, CA). Nonspecific interactions were blocked by using normal horse serum for 30 minutes at room temperature. Primary antibodies were diluted 1:20 in 0.01% phosphate buffered saline (PBS), pH 7.4 and incubated overnight at 4°C in a humidified chamber. Bound antibodies were detected by using a BioGenex IHC kit containing biotinylated secondary antibody and streptavidin-conjugated HRP enzyme coupled with 3,3′-diaminobenzidine chromagen solution in proprietary formulations (BioGenex, San Ramon, CA). Tissue was counterstained with hematoxylin, blued with saturated lithium carbonate solution, and imaged on Aperio ImageScope (Leica Biosystems, Buffalo Grove, IL). Only membrane staining was quantified for ILK or MT1-MMP and was scored as absent, weak, moderate, or strong. Cores scored as moderate or strong were combined as positive staining.

For immunofluorescence experiments, cells were subcultured on 22mm^2^ glass coverslips (coated as indicated), washed twice with ice-cold PBS, and fixed with 4% paraformaldehyde in 0.12M sucrose in PBS for 10 minutes at room temperature. Cells were blocked with 5% normal goat serum in PBS for 1 hour at room temperature, incubated with primary antibody (1:100) in 1% normal goat serum in PBS for 1h at 37°C, rinsed thrice for 5 minutes with PBS, and incubated with appropriate Alexa-Fluor conjugated secondary antibody at a 1:500 dilution for 30 minutes at 37°C. After washing, cells were allowed to dry, mounted with VECTASHIELD Mounting Media with 4′, 6-diamidino-2-phenylindole (DAPI) (Vector laboratories, Burlingame, CA), and visualized on an EVOS^®^ FL digital inverted fluorescence microscope (Advanced Microscopy Group, Mill Creek, WA).

### Formation of multicellular aggregates (MCAs) and analysis of cell spreading

MCAs were generated using a modification of the hanging drop method as previously described [56]. Briefly, cells were harvested in complete medium and diluted to a concentration of 1×10^5^ cells/ml. From this, 20μl of cell suspension was gently pipetted onto the underside of a 150mm tissue culture plate lid. To avoid dehydration of the hanging droplet, 20ml of sterile PBS was placed in the tissue culture dish immediately prior to inverting the droplet-containing lid. MCA formation was monitored after incubation at 37°C for 6-12 hours. To assess cell spreading, cells were plated in the presence of vehicle or QLT0267 and incubated for 12 h prior to visualization by light microscopy.

### Adhesion and invasion assays

To quantify cell adhesion to collagen I, tissue culture wells were coated with 10μg/ml collagen type I in sodium carbonate, pH 9.6 overnight at 4°C, washed with PBS, and air dried. Ovarian cancer cells were seeded as indicated, allowed to adhere at 37°C for 30 minutes, and washed for removal of non-adherent cells. Incubation media from each well was removed by suction pipette and wells were placed on a laboratory shaker. Room temperature PBS (2.5ml) was pipetted onto the wall of each well followed by shaking for 5 minutes. This procedure was repeated 5 times for each sample. After washing, cells were fixed and adherent cells were enumerated for analysis. Assays were performed in triplicate and five 20X fields/well were counted. For evaluation of adhesion to mesothelial cells, tissue culture wells were coated with 10μg/ml collagen type I in sodium carbonate, pH 9.6 overnight at 4°C, washed with PBS, and air dried. LP9 human peritoneal mesothelial cells were seeded and grown for 48 hours to form a tightly woven monolayer. Cancer cells were labeled with CellTracker™ Green, (5-Chloromethylfluorescein Diacetate (CMFDA), Life Technologies Inc, Carlsbad, CA) for 30 minutes at 37°C. The live mesothelial monolayer was washed twice with PBS, seeded with cells as indicated, and allowed to adhere for 30 minutes. Wells were then washed for removal of non-adherent cells as described above and fixed. Fluorescent cells were enumerated. Assays were performed in triplicate and five fields/well were counted.

To assess invasion, an 8μm microporous membrane located within the upper compartment of a transwell insert (BD Biosciences, San Diego, CA) was coated with 10μg/ml collagen type I in sodium carbonate, pH 9.6 overnight at 4°C, washed with PBS, and air dried. Cells were seeded atop the filter and the apparatus incubated at 37°C for 24 hours. After incubation, migrated cells passing through the 8μm pore filter were fixed, stained with Diff-Quik (Fisher Scientific, Pittsburgh, PA) and enumerated. All experiments were completed in triplicate and five fields/well were counted. A 3-dimensional organotypic meso-mimetic invasion assay was used to assess invasion of mesothelial cells overlaying a collagen matrix as described [57-58]. Briefly, a 3D collagen type I matrix was plated atop an 8μm microporous membrane within a transwell insert (BD Biosciences, San Diego, CA). The matrix was then overlaid with LP9 human peritoneal mesothelial cells and the mesothelial cells were allowed to grow to confluence, forming a tight monolayer. CellTracker™ Green-labelled cells were seeded atop the live monolayer and the co-culture was incubated at 37°C for 48 hours in a 1:1 ratio of complete media for each cell type. Labeled cells passing through the organotypic culture and the 8μm pore filter were fixed and enumerated. All experiments were completed in triplicate and five fields/well were counted.

### Statistical analyses

Statistical significance is defined as *p*<0.05 and was calculated employing a variety of statistical tests (Student's t-test, Mann-Whitney U Test, Kruskal-Wallis) as indicated using SigmaPlot v.12 (Systat Software Inc., San Jose, CA). Statistical tests were chosen based on the normality of the data set. Parametric tests (i.e. Student's t-test) were used to compare the means of two independent samples; however, since much of the data within these studies could not be completely described by two parameters (mean and standard deviation), non-parametric tests were utilized. The Kruskal-Wallis test, a non-parametric version of ANOVA, was used in instances where there were equal sample sizes in all groups and the comparatives had one nominal variable and one measurement variable. This test was employed to examine whether the mean ranks of the measurement variable were the same in all groups. Measurement observations were converted to their ranks in the overall data set; when scores received tied ranks, a correction factor was used. The Mann-Whitney U test, the non-parametric analogue to the Student's t-test, was also used in instances where the comparative had one nominal variable and one measurement variable, however this test was only utilized when comparing exactly two values.
